# Dataset on tip vortex formation noise produced by wall-mounted finite airfoils with sinusoidal and porous tip geometries

**DOI:** 10.1016/j.dib.2020.105471

**Published:** 2020-04-06

**Authors:** Tingyi Zhang, Danielle Moreau, Thomas Geyer, Jeoffrey Fischer, Con Doolan

**Affiliations:** a The University of New South Wales, Sydney, NSW 2052, Australia; b Brandenburg University of Technology Cottbus—Senftenberg, Cottbus 03046, Germany

**Keywords:** Aeroacoustics, Airfoil noise, Beamforming, Wingtip

## Abstract

Airfoil tip vortex formation noise is a significant noise source in many aerodynamic applications such as aircraft, fans, rotors and propellers. The data collection presented in this paper examines the effects of sinusoidal geometry and porosity on the tip vortex formation noise produced by finite length airfoils. The use of serrated and porous materials is inspired by silent owl-wings and is a promising approach to control flow-induced noise. Noise measurements have been taken using a 47-channel planar microphone array in the anechoic wind tunnel at the Brandenburg University of Technology. Over 2600 unique test cases with variations in sinusoidal tip geometry (amplitude and wavelength) and flat tip porosity were measured during the experimental campaign for a NACA0012 and NACA614 airfoil. The microphone data have been processed using acoustic beamforming software named Acoular to produce one-third-octave band tip noise spectra.

Specifications table

**Table:** 

Subject	Aerospace Engineering
Specific subject area	Acoustics, aerodynamics and fluid mechanics
Type of data	Tables in .csv format One-third-octave band acoustic spectra in .txt and .tif format
How data were acquired	Experiments were performed in the anechoic wind tunnel at the Brandenburg University of Technology in Cottbus, Germany. Acoustic measurements were obtained using a planar 47-channel microphone array. A National Instruments 24-bit multichannel measurement system combined with in-house software (written using a combination of Labview and Python codes) was used to record the microphone data.
Data format	Raw and analysed
Parameters for data collection	The test models were NACA0012 and NACA6412 airfoils with an aspect ratio of 2 and sinusoidal or porous flat tip geometries. Measurements were taken with natural and forced airfoil boundary layer transition at chord-based Reynolds numbers of 25,000 to 225,000 and geometric angles of attack −10° to 20°
Description of data collection	The data collection is an extended set of experimental measurements on wall-mounted finite airfoil tip vortex formation noise when employing novel sinusoidal and porous tip geometries.
Data source location	The University of New South Wales, Sydney, Australia 33° 55′ 4″ S, 151° 13′ 52″ E
Data accessibility	Repository name: Mendeley data Direct URL to data: http://dx.doi.org/10.17632/2jzkxbwgp8.1

## Value of the data

• This dataset provides new information on the effect of sinusoidal tip geometry and flat tip porosity on airfoil tip vortex formation noise.
• The data can be used to assist the development of low-noise airfoil tip devices using passive noise control.
• The data can be used to validate computational fluid dynamics and computational aeroacoustic simulations of different airfoil tip shapes.
• Students, researchers and those working in industry, who are interested in the acoustic behaviour of wall-mounted finite airfoils and specifically the wingtip, will benefit from this data collection.

## 1 Data

The data presented in this article is a set of tip vortex formation noise measurements taken for a wall-mounted finite airfoil employing novel sinusoidal and porous tip devices This dataset extends and complements the authors’ previous benchmark dataset on the tip vortex formation noise produced by a wall-mounted finite airfoil with flat and rounded tip geometries [1].

**Fig. 1:**
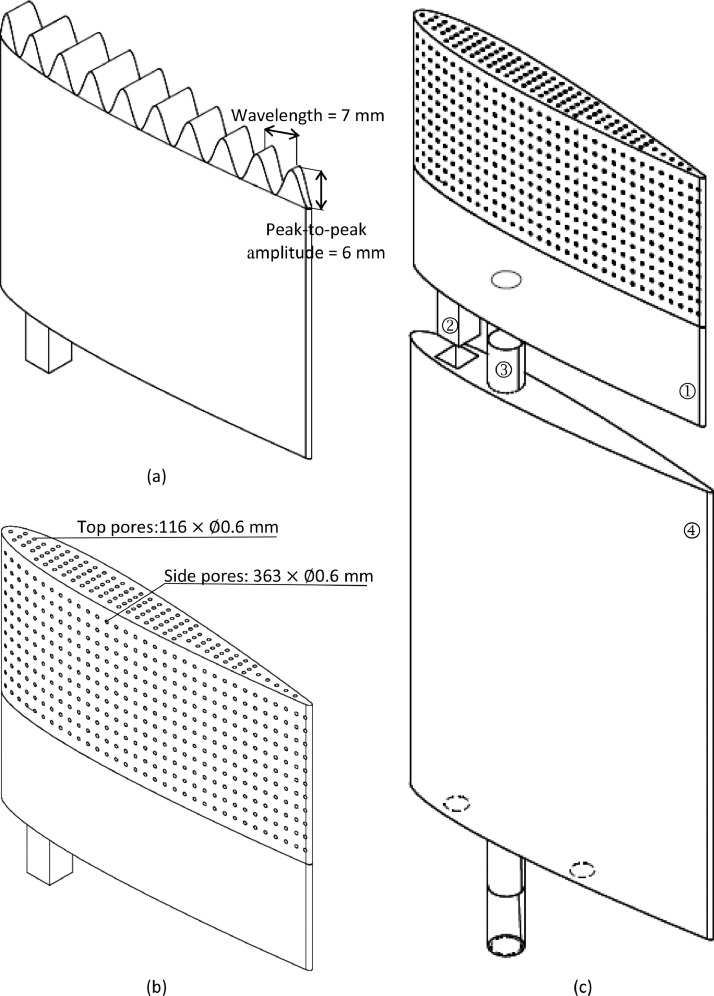
Interchangeable tips and assembled airfoil model. (a) Sinusoidal interchangeable tip (A3W10). (b) Porous interchangeable tip (P1, porosity = 13.4%). (c) Schematic assembly of a NACA0012 base model, porous interchangeable tip (P1) and central shaft. ① interchangeable tip, ② self-lock mechanism, ③ central shaft and ④ base model.

The dataset contains raw acoustic pressure time histories (.h5 format) and one-third-octave band sound pressure level spectra (.txt and .tif format) processed using Acoular. Tables 1 and 2 give detailed geometrical parameters of the sinusoidal and porous tips, respectively. Table 3 states the position of each microphone capsule in the array. Table 4 is a test matrix of the entire acoustic measurement campaign. Table 5 gives one-third-octave band tip noise sound pressure levels for a tripped NACA0012 airfoil with a porous tip at geometric angles of attack of *α* = 0: 5: 20°, and a Reynolds number of *Re_C_* = 2.25 × 10^5^, based on chord. Fig. 1 shows examples of the interchangeable airfoil tip design and how different parts of the airfoil model are assembled. Fig. 4 shows the tip noise spectra for tripped NACA0012 and NACA6412 airfoils with sinusoidal and porous tips at geometric angles of attack of *α* = 0° and 15° and a Reynolds number of *Re_C_* = 2.25 × 10^5^. Raw and processed data for each table and figure can be accessed via the direct URL to the data: http://dx.doi.org/10.17632/2jzkxbwgp8.1. The dataset for the reference NACA0012 and NACA6412 airfoil models with flat tip can be accessed via: http://dx.doi.org/10.17632/6x59x7x3ny.2 [1].

**Table 1:** Geometrical parameters of sinusoidal tips.

Airfoil profile	Tip geometry	Peak amplitude* (mm)	Wavelength (mm)	Number of chordwise wavelengths	Spanwise location of local minima (mm)
NACA0012	A3W10	3	7	10	134
	A5W10	5	7	10	130
	A10W10	10	7	10	120
	A3W5	3	14	5	134
	A5W5	5	14	5	130
	A10W5	10	14	5	120
	A3W3	3	23.3	3	134
	A5W3	5	23.3	3	130
	A10W3	10	23.3	3	120
	A3to10W5◊	3: 1.75: 10	14	5	127
	A10to3W5◊	10: −1.75: 3	14	5	127
NACA6412	A3W10	3	7	10	134
	A5W10	5	7	10	130
	A3W5	3	14	5	134
	A5W5	5	14	5	130
	A3to10W5◊	3: 1.75: 10	14	5	127
	A10to3W5◊	10: −1.75: 3	14	5	127

⁎ Peak amplitude is half of the peak-to-peak amplitude, shown in Fig. 1(a).

◊ The varying amplitude starts from the leading edge and ends at the trailing edge.

**Table 2:** Geometrical parameters of porous tips.

Airfoil profile	Tip geometry	Diameter of top pores (mm)	Diameter of side pores (mm)	Porosity (%)
NACA0012	P1	0.6	0.6	13.8
	P2	1	0.6	27.2
	P3	1	0.8	31.2
	P4	1.6	1.6	40.4
	P5	2	2	50.0
	P6∧	1	0	22.1
	P7∧	0	0.6	5.14
	P8□	1	1	22.9
NACA6412	P3	1	0.8	31.1
	P4	1.6	1.6	41.4
	P5	2	2	50.2

∧ A pore diameter of 0 indicates no perforated structure present on the top or side surface.

□ The porous structure for P8 covers only half of the tip extending from mid-chord to the trailing edge.

**Table 3:** Positions of the microphone capsules in the planar microphone array.

Microphone number	X (mm)	Y (mm)	Microphone number	X (mm)	Y (mm)	Microphone number	X (mm)	Y (mm)
0	−146	634	16	−193	−152	32	83	−42
1	−67	237	17	−120	−215	33	634	146
2	−377	530	18	−29	−244	34	237	67
3	−152	193	19	−463	−30	35	530	377
4	−551	345	20	−242	−139	36	193	152
5	−215	120	21	−88	−29	37	345	551
6	−641	108	22	−73	−269	38	120	215
7	−244	29	23	−42	−83	39	108	641
8	−30	463	24	67	−237	40	29	244
9	−139	242	25	152	−193	41	463	30
10	−29	88	26	215	−120	42	242	139
11	−349	307	27	641	−108	43	88	29
12	−269	73	28	244	−29	44	307	349
13	−83	42	29	139	−242	45	73	269
14	−634	−146	30	29	−88	46	42	83
15	−237	−67	31	269	−73

**Table 4:** Overview of experimental configurations.

Airfoil profile	Tip geometry	Geometric angle of attack (^∘^)+	Reynolds Number (‘000)+	Airfoil boundary layer transition type
*NACA0012*	A3W10	0: 5: 20	25: 25: 225	Natural and forced
	A5W10	0: 5: 20	25: 25: 225	Natural and forced
	A10W10	0: 5: 20	25: 25: 225	Natural and forced
	A3W5	0: 5: 20	25: 25: 225	Natural and forced
	A5W5	0: 5: 20	25: 25: 225	Natural and forced
	A10W5	0: 5: 20	25: 25: 225	Natural and forced
	A3W3	0: 5: 20	25: 25: 225	Natural and forced
	A5W3	0: 5: 20	25: 25: 225	Natural and forced
	A10W3	0: 5: 20	25: 25: 225	Natural and forced
	A3to10W5	0: 5: 20	25: 25: 225	Natural and forced
	A10to3W5	0: 5: 20	25: 25: 225	Natural and forced
	P1	0: 5: 20	25: 25: 225	Natural and forced
	P2	0: 5: 20	25: 25: 225	Natural and forced
	P3	0: 5: 20	25: 25: 225	Natural and forced
	P4	0: 5: 20	25: 25: 225	Natural and forced
	P5	0: 5: 20	25: 25: 225	Natural and forced
	P6	0: 5: 20	25: 25: 225	Natural and forced
	P7	0: 5: 20	25: 25: 225	Natural and forced
	P8	0: 5: 20	25: 25: 225	Natural and forced
*NACA6412*	A3W10	−10, 0: 5: 20	25: 25: 225	Natural and forced
	A5W10	−10, 0: 5: 20	25: 25: 225	Natural and forced
	A3W5	−10, 0: 5: 20	25: 25: 225	Natural and forced
	A5W5	−10, 0: 5: 20	25: 25: 225	Natural and forced
	A3to10W5	−10, 0: 5: 20	25: 25: 225	Natural and forced
	A10to3W5	−10, 0: 5: 20	25: 25: 225	Natural and forced
	P3	−10, 0: 5: 20	25: 25: 225	Natural and forced
	P4	−10, 0: 5: 20	25: 25: 225	Natural and forced
	P5	−10, 0: 5: 20	25: 25: 225	Natural and forced

+ Geometric angle of attack and Reynolds number are provided as an ascending arithmetic sequence of the form S : I: E, with values starting at S and ending at E in increments of I.

**Table 5:** One-third-octave band tip noise sound pressure levels for a tripped NACA0012 airfoil with P1 tip (porosity = 13.8%) at different geometric angles of attack and a Reynolds number of $Re_{C}=$ 2.25  ×  10^5^.

One-third octave band centre frequency (kHz)	One-third octave band tip noise sound pressure levels (dB re 20 μPa)				
	0^∘^	5^∘^	10^∘^	15^∘^	20^∘^
0.50	38.0	34.9	39.8	39.1	63.0
0.63	48.9	49.9	50.0	53.9	65.3
0.80	45.8	46.6	48.1	53.4	62.0
1.00	26.5	26.8	26.0	28.3	30.9
1.25	30.5	31.2	31.6	33.5	35.4
1.60	34.8	35.3	37.6	43.0	43.7
2.00	40.4	41.0	44.1	48.4	49.8
2.50	42.5	43.4	46.5	50.7	52.1
3.15	42.2	42.2	46.1	49.7	51.8
4.00	41.6	42.2	44.6	48.6	51.3
5.00	41.7	42.42	44.6	48.3	51.4
6.30	42.7	43.2	44.3	48.5	51.5
8.00	42.5	43.2	44.6	48.2	51.0
10.00	41.9	43.6	46.5	48.5	50.6
12.50	38.5	40.6	44.3	45.8	47.6
16.00	40.9	41.3	43.9	45.6	46.3
20.00	49.6	50.1	51.6	53.8	54.0

## 2 Experimental design, materials, and methods

The airfoils used in this experimental campaign have NACA0012 and NACA6412 profile with either a sinusoidal or porous tip (as listed in Tables 1 and 2, respectively, and shown in Fig. 1). Two base NACA0012 and NACA6412 models with a span of 90 mm were manufactured from aluminium using Computer Numerical Control (CNC) to support interchangeable tips. The interchangeable tips each have a span of 50 mm and were produced with the same airfoil profiles as the base models. The base models and interchangeable tips are assembled together with a self-lock mechanism along with a central shaft, as shown in Fig. 1(c). When assembled, the airfoils have a theoretical chord length of 70 mm, an actual chord length of 67 mm due to a truncated rounded trailing edge with diameter of 1.0 mm and a span of 140 mm, corresponding to an aspect ratio of 2.

The sinusoidal tips (shown in Fig. 1(a)) have amplitudes ranging from 3 to 10 mm and wavelengths between 7 and 23.3 mm (see Table 1) and were designed using a sinusoidal function. The sinusoidal tip thickness is equal to the local airfoil thickness along the camber line at each spanwise location. All sinusoidal tips were manufactured from aluminium using a computer numerically controlled machining process. Porous tips with porosities ranging from 5.14% to 50.2% (shown in Fig. 1(b)) were created by varying the positions and diameters of the pores (as given in Table 2). The porous interchangeable tips were created via 3D printing using resin [2,3]. The porous structure encompassed the outer 60% of the interchangeable tip. NACA0012 and NACA6412 porous tips were designed to have comparable porosity. The airfoil models were tested with both natural and forced boundary layer transition. For the latter case, 0.4 mm thick zig–zag tape (manufactured by Glasfaser Flugzeugservice) was applied at 10% chord on both sides of the assembled airfoil (as described in [1]) to suppress the laminar-instability tonal noise.

**Fig. 2:**
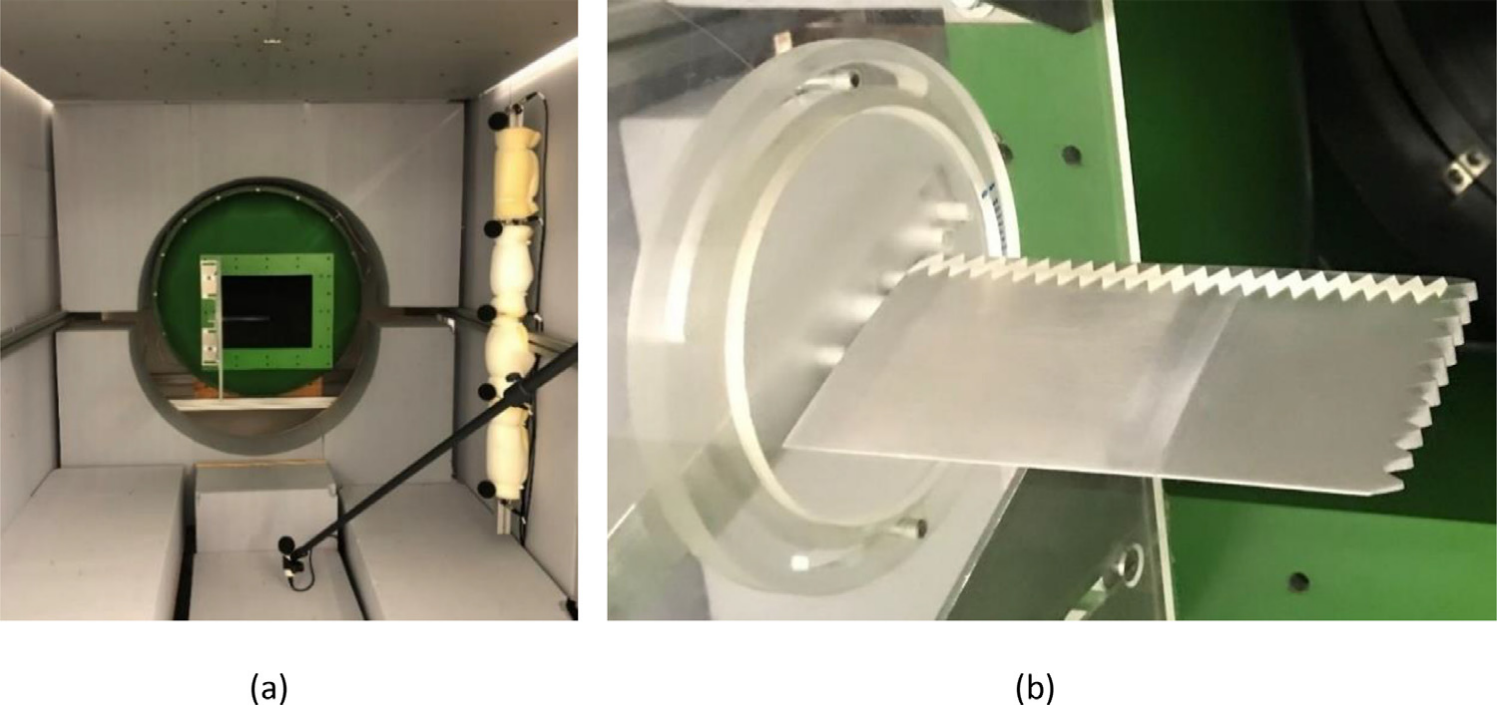
Experimental setup in the aeroacoustic wind tunnel. (a) Wind tunnel setup. Note that all the six anti-wind foam-covered microphones shown in (a) were not used in the measurements. (b) A tripped sinusoidal airfoil (A3W10) mounted on the side plate.

Acoustic measurements were taken in the open-jet aeroacoustic wind tunnel at the Brandenburg University of Technology in Cottbus, Germany (see Fig. 2(a)) [4]. Full details of this facility and the microphone array are given in [1]. As shown in Fig. 2, the assembled airfoil was mounted to a circular disk that was in turn inserted into a Perspex side plate of size 400 mm × 360 mm. The side plate was then flush mounted to the nozzle of size 280 mm × 230 mm. This side plate arrangement allowed the airfoil to be rotated around its half-chord location to achieve a range of angles of attack. The maximum axial turbulence intensity of the facility is in the order of 0.2% at a flow speed of 50 m/s.

Acoustic measurements were recorded with a planar 47-channel microphone array (see Fig. 2(a)) [1]. The flush-mounted microphones have a frequency range between 20 and 16,000 Hz and were located 710 mm above the trailing edge of the assembled model at zero angle of incidence. Forty seconds of microphone data were recorded for each measurement configuration at a sampling frequency of 51,200 Hz using a National Instruments 24-bit multichannel measurement system combined with in-house software.

**Fig. 3:**
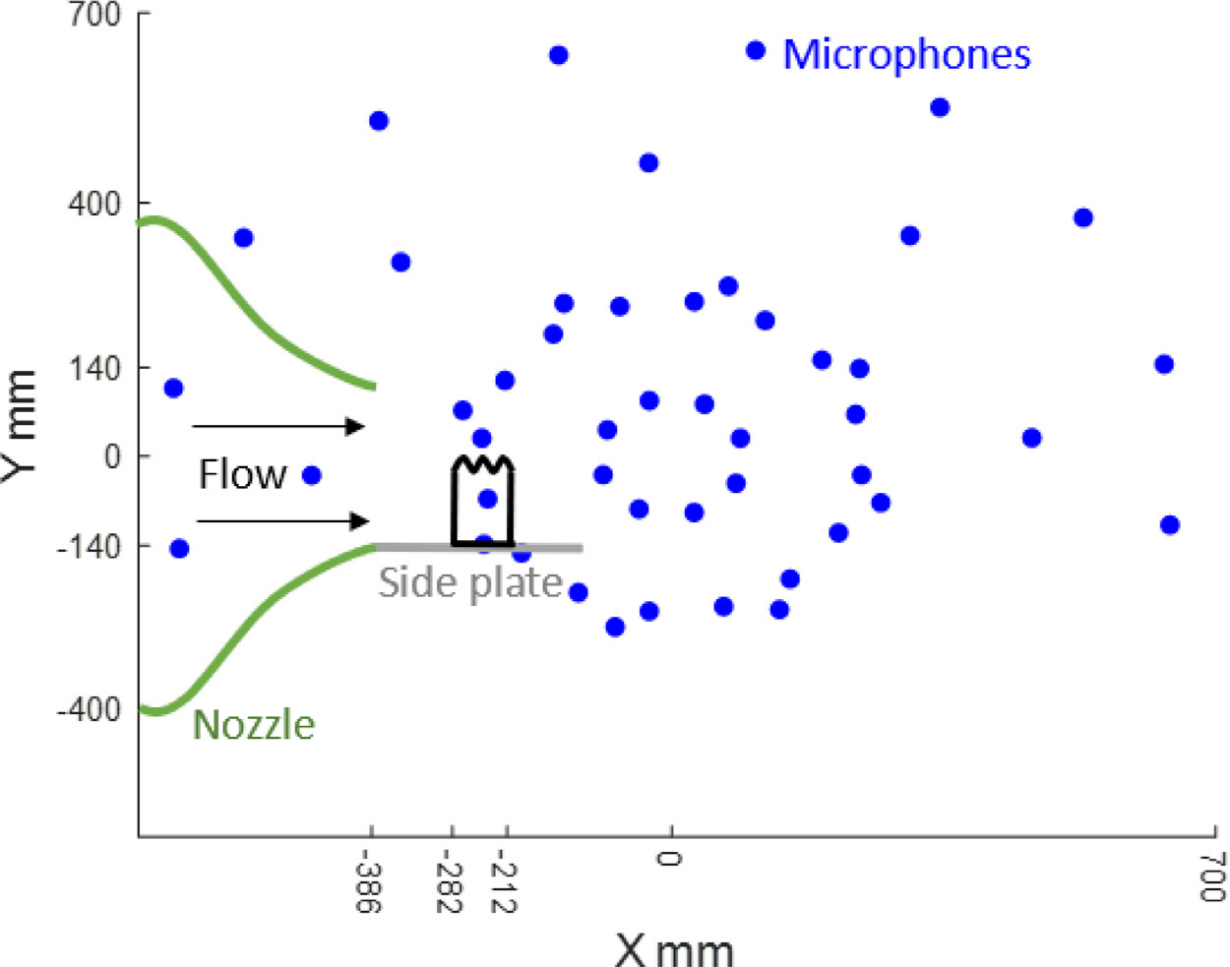
Schematic diagram of the nozzle, airfoil model (shown as a black rectangle with a sinusoidal tip) and planar microphone array.

The centre of the microphone array is the origin of the coordinate system. The streamwise direction is along the positive *X* axis, while the spanwise direction is along the positive *Y* axis. The positive *Z* axis points from the wind tunnel ceiling down towards the airfoil. The trailing edge of the airfoil at zero angle of incidence is 710 mm below the planar microphone array. The positions of the 47 microphones, from 0 to 46, are stated in Table 3. Fig. 3 is a schematic of the experimental setup.

A detailed test matrix is given in Table 4. Acoustic measurements for NACA0012 airfoil models were taken at geometric angles of attack between 0° and 20°, while an extra position of −10° was tested for all NACA6412 airfoil models. The geometric angle of attack can be converted to spanwise effective angle of attack using the procedure reported by Awasthi et al. [5]. All models were measured with both natural and forced transition at a range of Reynolds numbers from 2.5 × 10^4^ to 2.25 × 10^5^, based on chord.

**Fig. 4:**
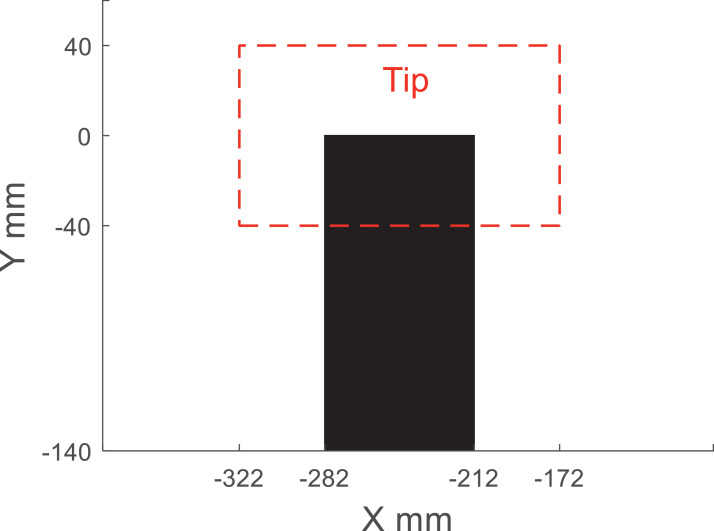
The integration region for the one-third-octave band spectra for the tip region (shown in red) and the whole airfoil (shaded in black).

**Fig. 5:**
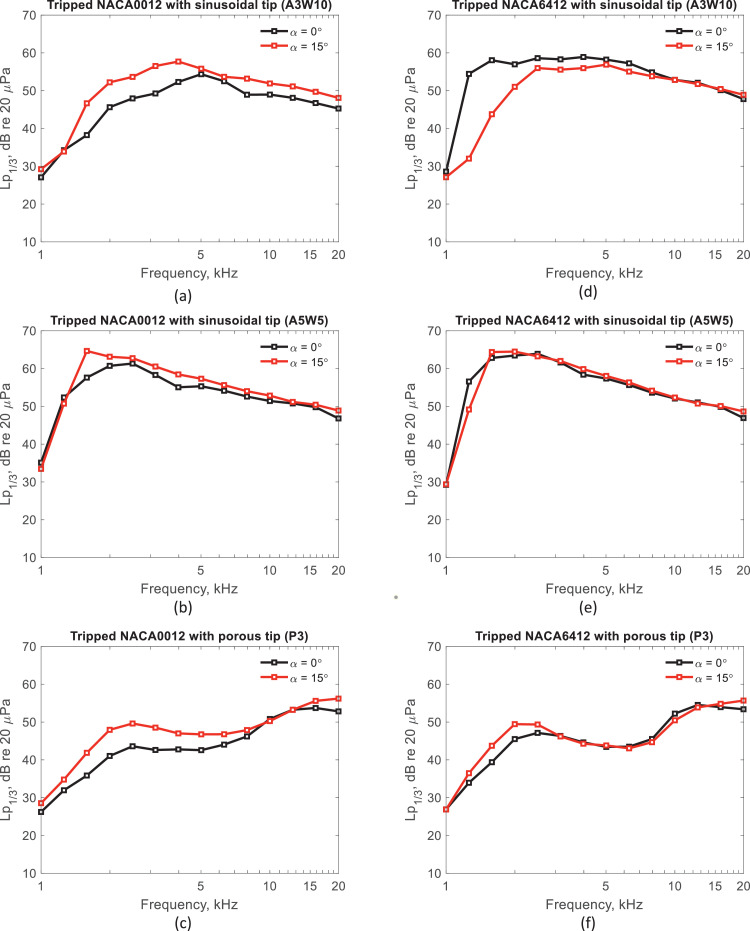
One-third-octave band tip noise spectra for the tripped airfoil at α=0^∘^ and 15^∘^ and $Re_{C}=\;$2.25  ×  10^5^. (a) to (c) NACA0012 airfoils with A3W10, A5W5 and porous P3 tip, respectively. (d) to (f) NACA6412 airfoils with A3W10, A5W5 and porous P3 tip, respectively.

Acoular [6] was used to process the raw acoustic data as detailed in [1]. The Cross-Spectral Matrix was obtained after a Fast Fourier Transformation. The CLEAN-SC deconvolution algorithm was used in the acoustic beamforming [7], [8], [9]. The integration region used to calculate the one-third-octave band acoustic spectra is shown in Fig. 4. The height of the tip integration region was defined to encompass 57% of the span and the width of this region is 214% of the chord. The size of the integration region was selected to ensure that the tip noise source was accurately captured based on inspection of the acoustic sound maps for the entire airfoil model. Table 5 gives an example of one-third-octave band tip noise sound pressure levels for a tripped NACA0012 airfoil with P1 tip at geometric angles of attack of *α* = 0: 5: 20° and a Reynolds number of $Re_{C}=$ 2.25  ×  10^5^. One-third-octave band tip noise spectra for tripped airfoils with sinusoidal and porous tips at $Re_{C}=$ 2.25  ×  10^5^ are also shown in Fig. 5.

## Appendix Supplementary materials

Image, application 1
